# Inconsistent primary motor cortex glucose hypometabolism in primary lateral sclerosis

**DOI:** 10.1007/s00415-025-13089-x

**Published:** 2025-05-20

**Authors:** Annaliis Lehto, Julia Schumacher, Jens Kurth, Bernd J. Krause, Elisabeth Kasper, Stefan Teipel, Johannes Prudlo, for the Alzheimer’s Disease Neuroimaging Initiative

**Affiliations:** 1 Translational Neurodegeneration Section “Albrecht Kossel”, Department of Neurology, Rostock University Medical Center, Rostock, Germany; 2 Deutsches Zentrum für Neurodegenerative Erkrankungen (DZNE), Rostock-Greifswald, Rostock, Germany; 3 Department of Neurology, Rostock University Medical Center, Rostock, Germany; 4 Department of Nuclear Medicine, Rostock University Medical Center, Rostock, Germany; 5 Department of Psychosomatic Medicine, Rostock University Medical Center, Rostock, Germany

**Keywords:** Primary lateral sclerosis, Cerebral glucose metabolism, FDG-PET, Primary motor cortex

## Abstract

**Objective:**

Primary lateral sclerosis (PLS) is a rare neurodegenerative disorder and a model for upper motor neuron degeneration, which is believed to begin in the primary motor cortex. However, clinical observation suggests that not all PLS cases show primary motor cortex glucose hypometabolism on 2-deoxy-2-[^18^F]fluoro-d-glucose positron emission tomography (FDG-PET). We aimed to assess the reliability of FDG-PET in identifying motor cortex hypometabolism over disease course in a sample of patients with PLS.

**Methods:**

Baseline FDG-PET data from nine consecutive PLS patients were analyzed. At least one follow up assessment was available for five patients. We extracted the average FDG-PET signal in the primary motor cortex and other motor regions and calculated the covariate-corrected *z* scores based on data of healthy controls from the Alzheimer’s Disease Neuroimaging Initiative (ADNI) cohort.

**Results:**

Among the nine patients evaluated, only four demonstrated glucose hypometabolism in the primary motor cortex across available baseline and follow up assessments. Glucose metabolism of motor regions declined over time in some patients, whereas others maintained stable levels despite a worsening in symptom severity.

**Interpretation:**

Primary motor cortex hypometabolism in PLS patients is less consistent than previously reported, and the absence of this hypometabolic sign should not be considered as irrefutable evidence against PLS in the diagnostic process. The findings of our study underline the heterogeneity of PLS, indicating that more precise diagnostic tools would be beneficial to confirm a PLS diagnosis at an earlier stage.

**Supplementary Information:**

The online version contains supplementary material available at 10.1007/s00415-025-13089-x.

## Introduction

Primary lateral sclerosis (PLS) is a rare neurodegenerative bulbospinal upper motor neuron syndrome [1, 2]. Although most cases of clinical PLS have cortical ubiquitin and TAR DNA-binding protein 43 (TDP-43) pathology [3, 4] and the PLS phenotype may appear as part of the phenotypic spectrum in familiar ALS pedigrees [5], PLS is no more considered part of the ALS spectrum by the Gold Coast Criteria [6]. The reasons for this are the lack of lower motor neuron signs and a significantly slower rate of progression when compared with classic ALS. Mills’ syndrome is a unilateral upper motor neuron disease, which appears clinically in the form of a slow progressive spastic hemiparesis; it is a rare variant of PLS [7, 8]. Despite the upper motor neuron impairment being the defining characteristic of PLS, radiological evidence suggests that PLS patients may also show widespread grey or white matter alterations beyond the cortical regions of motor control and the corticospinal tracts [9, 10]. In contrast to classic ALS, diagnosing PLS is challenging, especially in the first two years of the disease, as it often requires diverse and targeted investigations to exclude a multitude of other diseases (e.g. inflammatory central nervous system disorders, compressive myelopathy) and to distinguish it from mimics such as upper motor neuron-dominant ALS, hereditary spastic paraplegia (HSP), and progressive supranuclear palsy (PSP) [11–13]. Sensitive diagnostic biomarkers of PLS are still lacking and further diagnostic tools would be valuable.

PLS is a disease model for upper motor neuron degeneration, apparently beginning in the primary motor cortex (M1), which is why glucose hypometabolism in the precentral cortex would be expected to be detectable on a 2-deoxy-2-[^18^F]fluoro-d-glucose-positron-emission tomography (FDG-PET) scan. Indeed, some case reports support this notion [14–17], encouraging the utilization of an FDG-PET assessment in the differential diagnosis. However, clinical observations have revealed this not always to be the case, which prompted this study to investigate M1 hypometabolism in a sample of nine PLS cases both cross-sectionally and longitudinally. The main aim of the current study was to examine the reliability of FDG-PET in identifying M1 hypometablism in a consecutive sample of patients who fit the diagnostic criteria for PLS. We broadened our scope to include further motor regions, motivated by previous reports of structural changes in these wider areas [18, 19].

## Methods

### Participants

Our sample consisted of nine consecutive patients recruited via the Department of Neurology of the Rostock University Medical Centre who met the consensus diagnostic criteria for probable or definite PLS [1]. All patients completed at least one clinical and FDG-PET imaging assessment. Furthermore, all patients underwent a routine magnetic resonance imaging (MRI) with fluid-attenuated inversion recovery, T1-weighted and T2-weighted protocols to rule out alternative pathologies. Six of the nine patients went through genetic testing of the common ALS/PLS-causing genes linked to ALS. One out of the nine had an ALS/PLS-causing mutation, five were screened negative, and no genetic data was available for three cases. The ALS Functional Rating Scale Revised (ALSFRS-r) was administered and subdomain scores were calculated (items 1–3 for bulbar score, items 4–6 for upper limb score, items 8 and 9 for lower limb score).

Furthermore, the baseline FDG-PET images of 70 healthy controls (HCs) from the Alzheimer’s Disease Neuroimaging Initiative (ADNI) database (adni.loni.usc.edu) as described in a previous study [20] were included in the analysis. The ADNI was launched in 2003 as a public–private partnership, led by Principal Investigator Michael W. Weiner, MD. The primary goal of ADNI has been to test whether serial magnetic resonance imaging, PET, other biological markers, and clinical and neuropsychological assessment can be combined to measure the progression of mild cognitive impairment and early Alzheimer’s disease. For up-to-date information, see www.adni-info.org.

### FDG-PET acquisition and preprocessing

FDG-PET data of HC were obtained in preprocessed form from the ADNI database. These images were acquired using various scanners, following platform-specific protocols. To ensure consistency across different scanners, all original ADNI FDG-PET scans underwent standardized preprocessing steps. Comprehensive details about the acquisition and preprocessing of FDG-PET images can be found on the ADNI website (https://adni.loni.usc.edu/data-samples/adni-data/neuroimaging/pet/).

The PLS patients underwent dynamic PET imaging (4 × 5 min) of the brain using a Gemini TF 16 scanner (Philips Healthcare) at 30 min after the injection of 199 ± 18 MBq FDG. Prior to PET imaging, an auxiliary CT scan (120 kVp, 30 mAs) was performed. Four dynamic PET frames were recorded, examined for head movements and any conspicuous frames were excluded. A static PET data set was reconstructed using the manufacturer’s proprietary BLOB-OS reconstruction algorithm (3 iterations, 31 subsets), which was corrected for randoms, scatter, decay, and attenuation using information from the auxiliary CT. To match the spatial resolution of 6 mm of the ADNI data, the FDG-PET scans of the PLS patients were filtered to the identical resolution using a Butterworth filter. All FDG-PET scans were skull-stripped, intensity normalized using the scan average, and registered to an FDG-PET template [21]. No partial volume correction was applied to the FDG-PET scans.

### Regions of interest (ROIs)

For all participants, ROI masks were used to extract the average regional FDG-PET signal. The ROIs included: the primary motor cortex (M1), dorsal and ventral premotor cortex (PMd, PMv), supplementary motor area (SMA), and pre-supplementary motor area (pre-SMA) from the Human Motor Area Template [22] and the upper limb, lower limb, trunk, head and face, tongue and larynx regions from the Brainnetome Atlas [23].

### Statistical analysis

The statistical analyses were completed and figures were created in R (https://www.r-project.org/). To determine whether the PLS patients had extreme values of FDG uptake in investigated ROIs, the mean signal intensity of each ROI was expressed as a w-score, which represents a covariate-adjusted z score derived from a control group distribution. The procedure involved two main steps. First, the FDG signal for each ROI in the HCs was regressed against age and sex, yielding regression coefficients and residuals. These regression coefficients were subsequently applied to calculate residual values for the PLS patients. Second, the mean and standard deviation (SD) of the residuals in the HC group were utilized to calculate the respective *w* scores for the patient residuals. This adjustment enabled the identification of disease-related alterations in FDG uptake while controlling for age and sex differences. *W* scores follow the *z* distribution and are therefore directly translatable to *p* values. A *w* score < − 1.65 indicates a one-tailed *p* value below 0.05 and a *w* score < − 1.96 indicates a one-tailed *p* value below 0.025. For a longitudinal assessment, *w* scores were also determined for available follow up (FU) visits. *W* scores of all assessed regions for all patients can be found in Supplementary Table 1.

## Results

### Demographic and clinical data

A multifaceted characterization of our sample can be found in Table 1. The patients (*N* = 9) varied greatly in their age (mean = 61 ± 8 years) and disease duration at baseline (mean = 40 ± 24 months). Five patients (44%) displayed pseudobulbar affect (PBA). 44% of the patients were female when compared with 54% of the HCs. The HCs were on average older than the PLS patients (mean = 72 ± 5 years).

**Table 1:** Demographic and clinical characteristics of the PLS patients

Patients	Age at B	Sex	Probable vs definite PLS	Disease duration at B (months)	Onset region	Sympto-matic regions at BL	Bulbar score (max 12)	Upper limb score (max 12)	Lower limb score (max 8)	ALSFRS-r total (max 48)	PBA	Genetics	Whole M1 hypomet. at BL	BL to FU1 (months)	Whole M1 hypo-met. at FU1	FU1 to FU2 (months)	Whole M1 hypo-met. at FU2
Pat1	47	Female	Definite	54	Pseudobulbar	BUL, UL, LL	7	5	3	28	Yes	Negative	No	47	Bilateral	24	Bilateral
Pat2	76	Female	Definite	14	Pseudobulbar	BUL				42	Yes	Negative	No	12	No	22	No
Pat3	63	Male	Probable	7	Left lower limb	LL, UL				40	No	TBK1	Right hemisphere	17	Bilateral		
Pat4	62	Female	Definite	21	Pseudobulbar	BUL, UL, LL	8	8	3	34	Yes	Not tested	Left hemisphere	11	Left hemisphere		
Pat5	58	Female	Probable	26	Left lower limb	LL, UL, BUL	8	8	3	33	No	Negative	No	15	No		
Pat6	66	Male	Definite	39	Right lower limb	LL				35	No	Negative	No				
Pat7	55	Male	Definite	56	Pseudobulbar	BUL, UL, LL	8	10	8	41	No	Not tested	No				
Pat8	65	Male	Definite	65	Bilateral lower limb	LL, UL, BUL	8	4	2	25	Yes	Negative	No				
Pat9	58	Male	Definite	74	Left upper limb	LL, UL, BUL				31	Yes	Not tested	No				

Probable vs definite PLS pertains to the last available follow up of the patient and not to the classification at baseline. Bulbar score is the sum of items 1–3, the upper limb score is the sum of items 4–6 and lower limb score is the sum of items 8–9 of the ALSFRS-r. A *w* value below − 1.96 indicated hypometabolism. More information on *w* scores and their corresponding p values can be found in the methods section

*ALSFRS-r* ALS Functional Rating Scale Revised, *BL* baseline, *BUL* bulbar, *FU* follow up, *hypomet* hypometabolism, *L* left, *LL* lower limb, *M1* primary motor cortex, *R* right, *PBA* pseudobulbar affect, *TBK1* mutation in the TANK-binding kinase 1 gene, *UL* upper limb

Two patients (pat3 and pat9) initially showed slowly progressive unilateral upper motor neuron signs, corresponding to Mills’ syndrome [7, 16, 24, 25]. The symptoms started in the left lower limb for patient 3 and the right lower limb for patient 6. Over a period of 3 years, patient 3’s clinical signs spread from the left leg to the left hand, then to the right leg, right hand, and lastly to the bulbar region. Patient 6’s clinical signs spread over the course of 5 years from the right leg to the left leg, then the right hand and the left hand. Although both patients became bilaterally affected, the impairments remained asymmetrically more severe on the onset side of the body. Neither patient 3 nor patient 6 showed lower motor neuron signs on the clinical or electrodiagnostic assessments. Patient 3 showed asymmetric frontotemporal atrophy in the right hemisphere, whereas patient 6 showed light nonspecific atrophy. The cerebrospinal fluid examination and further investigations did not reveal other pathologies. The genetic analysis was negative for pat6, but a mutation in the TPK1-gene of uncertain significance was discovered in patient 3.

At the baseline assessment, six patients of our sample already had a symptomatic involvement of all three regions (bulbar, upper limb, and lower limb). For all the following results, right and left pertain to respective hemispheres.

### FDG-PET data

**Fig. 1:**
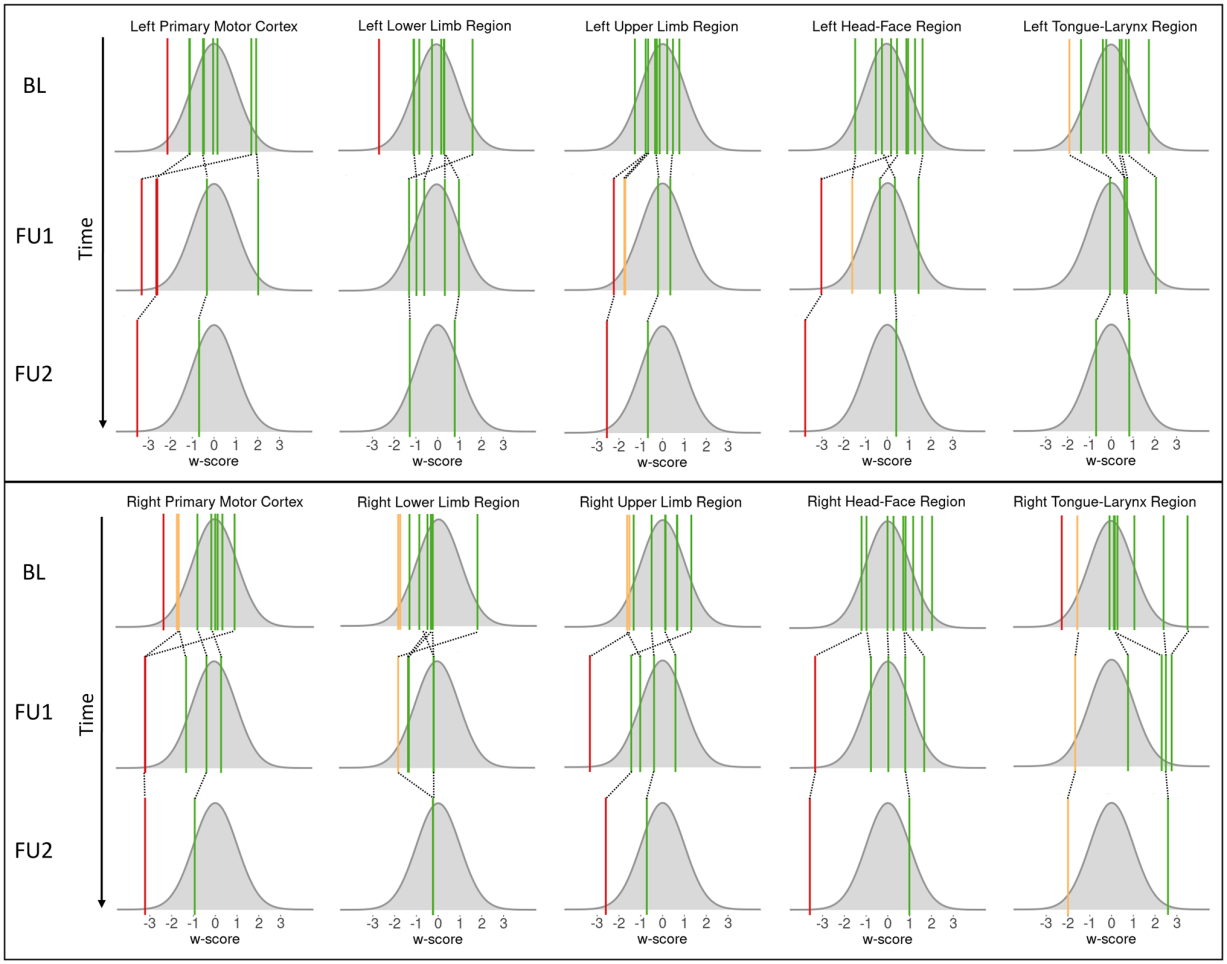
Regional *w* scores of PLS patients for available time points. The *w* scores of PLS patients are visualized with colored lines over a normal distribution. *W* scores > − 1.65 are shown in green (one-sided *p* value > 0.05), *w* scores between − 1.65 and − 1.96 are shown in yellow (one-sided *p* values between 0.05 and 0.025), and *w* scores < − 1.96 are shown in red (one-sided *p* value < 0.025). Right and left refer to the assessed hemisphere. *BL* baseline, *FU* follow up

Despite the considerable symptomatic burden, only four of the patients showed regional hypometabolism (w scores < − 1.96) in motor regions (whole M1 or a subregion of M1): patients 3, 4 and 6 from baseline onwards and patient 1 only from the first FU onwards (Table 1). The patients’ regional *w* scores have been projected onto the standard normal distributions in Fig. 1.

Despite the bulbar, upper limb, and lower limb involvement, all regional values of patient 1 stayed above *w* = − 2 at the baseline assessment. At FU assessments, patient 1 showed a bilateral decrease in the FDG uptake in M1 (right hemisphere FU1 *w* = − 3.2 to FU2 *w* = − 3.2, and left hemisphere FU1 *w* = − 3.3 to FU2 *w* = − 3.5). Patient 1 also had low *w* scores for the head and face region of M1 (right FU1 *w* = − 3.3 to FU2 *w* = − 3.6, and left FU1 *w* = − 3.0 to FU2 *w* = − 3.8) and for the upper limb region (right FU2 *w* = − 2.6, and left FU1 *w* = − 2.2 to FU2 *w* = − 2.6). Furthermore, patient 1 showed low FDG uptake in the left SMA (FU1 *w* = − 2.3 to FU2 *w* = − 2.9), the left pre-SMA (FU2 *w* = − 2.1), and the PMv (right FU1 *w* = − 3.4 to FU2 *w* = − 4.2, and left FU1 *w* = − 2.9 to FU2 *w* = − 4.2).

Patient 3 had asymmetric lower and upper limb involvement at baseline (Mills’ syndrome), and at FU1 the bulbar region had also become symptomatic. At baseline, patient 3 showed a decrease in the FDG uptake in the right M1 (*w* = − 2.3). At FU1, the *w* scores were low for the bilateral M1 (right *w* = − 3.2 and left *w* = − 2.6), the right upper limb region (*w* = − 3.3), the right SMA (*w* = − 3.1), and the right PMd (*w* = − 2.4).

At baseline, patient 4 had symptoms in the bulbar region, lower, and upper limbs, and the FDG uptake was low in the left M1 at both baseline (*w* = − 2.1) and at FU1 (*w* = − 2.6).

The baseline scan of patient 6 with lower limb involvement showed low *w* scores for the left lower limb region (*w* = − 2.6) and for the right tongue and larynx region (*w* = − 2.3). Furthermore, patient 6 had low FDG uptake in the bilateral SMA (right *w* = − 2.1 and left *w* = − 3.4), and left PMd (*w* = − 2.6).

The regional scores of the remaining five patients were all > − 1.96. *W* scores of all assessed regions for all patients can be found in Supplementary Table 1.

## Discussion

We aimed to assess the reliability of FDG-PET in identifying UMN degeneration in a consecutive sample of PLS patients. Among the nine patients evaluated, only four demonstrated glucose hypometabolism in the primary motor cortex across available baseline and follow up assessments. Of those four patients, three also exhibited glucose hypometabolism in motor regions beyond the primary motor cortex. Glucose metabolism of motor regions declined over time in some patients, whereas others maintained stable metabolic levels despite worsening symptom severity. No consistent relationships were observed between glucose metabolism levels and clinical variables such as age, disease duration, or symptom distribution. These findings suggest that the primary motor cortex hypometabolism in PLS patients is less consistent than previous case reports have indicated [14–16]. Consequently, the absence of this hypometabolic sign should not be considered as irrefutable evidence against a diagnosis of PLS.

Glucose metabolism is closely linked to neural activity and the observed interindividual variability in our findings may have several possible explanations. One reason could be that the cortical changes in the M1 are not conspicuous in all patients that meet the PLS diagnostic criteria. Although a loss of Betz cells in the primary motor cortex is reported in many autopsy studies [4], multiple PLS cases have also revealed corticospinal tract degeneration without a discernible loss of Betz cells [26]. MRI studies have revealed precentral gyrus atrophy in PLS when compared with healthy controls on the group level [27–29] but also clearly highlighted its progressive nature over the disease course for individual patients [18, 30]. FDG-PET may not be sensitive enough to identify limited neurodegeneration in the first stages of the disease in each PLS patient.

Further between-patient variability in glucose metabolism in our sample may be related to differences in the underlying pathology. Because no autopsy data are available for the patients in our sample, the pathologies leading to the clinical phenotypes meeting the PLS criteria cannot be determined. A renowned autopsy report on patients with PLS phenotype describes a degeneration of the M1 and corticospinal tracts, numerous TDP-43 inclusions in the M1, limited or no inclusions in the lower motor neurons and further inclusions in extramotor neocortex [3, 4]. Nevertheless, also other pathologies, such as tau can lead to the clinical phenotype of PLS [12, 31]. A recent clinico-pathological case series of patients that filled the PLS diagnostic criteria highlighted both the pathological variability that may lead to this phenotype and the difficulties of differential diagnosis [32].

One further reason for the variability in our sample could be a heterogeneous spread of PLS pathology: it may be that the pathological spread is not only anterograde or “dying forward” (whereby degeneration spreads from the soma of the Betz cell to the distal axonal parts of the upper motor neuron) [33, 34] but also retrograde or “dying back” (in which degeneration spreads from the distal axonal parts of the upper motor neuron to the soma of the Betz cell) [35]. For instance, about 50% of PLS patients exhibit a symmetric ascending pattern of paralysis (from the lower limbs to the upper limbs to the bulbar regions), which supports the hypothesis of length-dependent dying back of corticospinal axons, whereas other patients show a more asymmetric or “patchy” spread of symptoms [36].

Finally, the precise cellular source of the FDG-PET signal remains undefined. Beyond neuronal glucose consumption, FDG uptake in cerebral tissue is dependent on astrocytes [37] and microglia [38]. A comprehensive overview of the relative contributions of these cell types to the FDG signal has yet to be provided. Previous PET studies in PLS have shown that both neuronal death [39] and glial activation, which co-localizes with grey matter atrophy [40, 41], exert opposing effects on the FDG-PET signal. Therefore, further investigations are required to clarify the cellular and molecular mechanisms underlying FDG signal changes in individual patients, providing a deeper understanding of disease pathology and its heterogeneity.

The diagnosis of PLS remains challenging, particularly its differentiation from UMN-dominant ALS, PSP and HSP [11, 13]. So far, no specific diagnostic biomarkers exist to enhance the diagnostic accuracy of PLS, necessitating a broad array of assessments including magnetic resonance imaging and transcranial magnetic stimulation [13, 32]. FDG-PET has been proposed as a potentially sensitive diagnostic tool; however, our findings suggest that its utility in clinical practice for diagnosing PLS may be limited and unreliable.

Our study is not without limitations. Like many other PLS studies, our sample size was small due to the rarity of this disease. Furthermore, in our sample, the HCs were older than the patients. Previous publications have reported primary motor cortex FDG-PET signal to remain relatively unaffected by age-related change [42] and found only a moderate decrease over the span of 40–85 years [43]. To minimize the possible age effect on the FDG-PET signal in our sample, we utilized covariate-adjusted *w* scores. In addition, the subregions of the primary motor cortex included in our analysis were relatively small and the spatial resolution of the imaging data (6 mm) introduced potential partial volume effects. Such effects may have led to spill-in signals from neighboring regions, potentially confounding the true FDG uptake in smaller areas and hindering the detection of localized metabolic changes. To identify subtle, small-scale changes, PET cameras with higher spatial resolution should be considered in future investigations.

In conclusion, FDG-PET does not appear to be a reliable diagnostic tool for PLS, particularly in the early disease stages when a sensitive and reliable biomarker would be most beneficial. Therefore, caution should be exercised when using FDG-PET as an additional diagnostic tool in suspected cases of PLS. Despite the challenges, there is an increasing interest in the delineation of the clinical entity of PLS. The findings of our study underline the heterogeneity within the patients that meet the diagnostic criteria of PLS. Future studies with larger cohorts are needed to validate the present findings and to investigate the clinical and biological features underlying this individual variability in PLS.

## Supplementary Information

Below is the link to the electronic supplementary material.

Supplementary file1 (PDF 415 kb)

## Data Availability

The data analyzed in the current study can be received from the corresponding author upon reasonable request from qualified investigators.

## References

[1] Turner MR, Barohn RJ, Corcia P et al (2020) Primary lateral sclerosis: consensus diagnostic criteria. J Neurol Neurosurg Psychiatry 91:373–377. 10.1136/jnnp-2019-322541 32029539 10.1136/jnnp-2019-322541 PMC7147236

[2] Bede P, Pradat P-F, Lope J et al (2022) Primary lateral sclerosis: clinical, radiological and molecular features. Rev Neurol (Paris) 178:196–205. 10.1016/j.neurol.2021.04.008 34243936 10.1016/j.neurol.2021.04.008

[3] Mackenzie IRA, Briemberg H (2020) TDP-43 pathology in primary lateral sclerosis. Amyotroph Lateral Scler Front Degener 21:52–58. 10.1080/21678421.2020.1790607 10.1080/21678421.2020.1790607 32657153

[4] Mackenzie IRA (2020) Neuropathology of primary lateral sclerosis. Amyotroph Lateral Scler Front Degener 21:47–51. 10.1080/21678421.2020.1837173 10.1080/21678421.2020.1837173 33602010

[5] Gómez-Tortosa E, Van der Zee J, Ruggiero M et al (2017) Familial primary lateral sclerosis or dementia associated with Arg573Gly TBK1 mutation. J Neurol Neurosurg Psychiatry 88:996–997. 10.1136/jnnp-2016-315250 28365590 10.1136/jnnp-2016-315250

[6] Shefner JM, Al-Chalabi A, Baker MR et al (2020) A proposal for new diagnostic criteria for ALS. Clin Neurophysiol Off J Int Fed Clin Neurophysiol 131:1975–1978. 10.1016/j.clinph.2020.04.005 10.1016/j.clinph.2020.04.005 32387049

[7] Mills CK (1900) A case of unilateral progressive ascending paralysis, probably representing a new form of degenerative disease. J Nerv Ment Dis 27:195

[8] Jaiser SR, Mitra D, Williams TL, Baker MR (2019) Mills’ syndrome revisited. J Neurol 266:667–679. 10.1007/s00415-019-09186-3 30631918 10.1007/s00415-019-09186-3 PMC6394692

[9] Finegan E, Li Hi Shing S, Chipika RH et al (2019) Widespread subcortical grey matter degeneration in primary lateral sclerosis: a multimodal imaging study with genetic profiling. NeuroImage Clin 24:102089. 10.1016/j.nicl.2019.102089 31795059 10.1016/j.nicl.2019.102089 PMC6978214

[10] Finegan E, Shing SLH, Chipika RH et al (2021) Extra-motor cerebral changes and manifestations in primary lateral sclerosis. Brain Imaging Behav 15:2283–2296. 10.1007/s11682-020-00421-4 33409820 10.1007/s11682-020-00421-4

[11] Gordon PH, Cheng B, Katz IB et al (2009) Clinical features that distinguish PLS, upper motor neuron-dominant ALS, and typical ALS. Neurology 72:1948–1952. 10.1212/WNL.0b013e3181a8269b 19487653 10.1212/WNL.0b013e3181a8269b

[12] Nagao S, Yokota O, Nanba R et al (2012) Progressive supranuclear palsy presenting as primary lateral sclerosis but lacking parkinsonism, gaze palsy, aphasia, or dementia. J Neurol Sci 323:147–153. 10.1016/j.jns.2012.09.005 23026537 10.1016/j.jns.2012.09.005

[13] Fullam T, Statland J (2021) Upper motor neuron disorders: primary lateral sclerosis, upper motor neuron dominant amyotrophic lateral sclerosis, and hereditary spastic paraplegia. Brain Sci 11:611. 10.3390/brainsci11050611 34064596 10.3390/brainsci11050611 PMC8151104

[14] Cosgrove J, Jamieson S, Chowdhury FU (2015) Teaching NeuroImages: hypometabolism of the primary motor cortex in primary lateral sclerosis. Neurology 84:e206–e206. 10.1212/WNL.0000000000001688 26078407 10.1212/WNL.0000000000001688

[15] Claassen DO, Josephs KA, Peller PJ (2010) The stripe of primary lateral sclerosis: focal primary motor cortex hypometabolism seen on fluorodeoxyglucose F18 positron emission tomography. Arch Neurol 67:122–125. 10.1001/archneurol.2009.298 20065142 10.1001/archneurol.2009.298

[16] Laere KV, Wilms G, Damme PV (2016) FDG-PET findings in three cases of Mills’ syndrome. J Neurol Neurosurg Psychiatry 87:222–223. 10.1136/jnnp-2014-309952 25922081 10.1136/jnnp-2014-309952 PMC4752632

[17] Lisei Coscia D, García Lucero ME, Zeidan Ramón N (2022) Primary lateral sclerosis: diagnostic contribution of brain [18F]FDG PET/CT. Rev Espanola Med Nucl E Imagen Mol 41:124–125. 10.1016/j.remnie.2021.04.012 10.1016/j.remnie.2021.04.012 35292140

[18] Tahedl M, Tan EL, Shing SLH et al (2023) Not a benign motor neuron disease: longitudinal imaging captures relentless motor connectome disintegration in primary lateral sclerosis. Eur J Neurol 30:1232–1245. 10.1111/ene.15725 36739888 10.1111/ene.15725

[19] Pioro EP, Turner MR, Bede P (2020) Neuroimaging in primary lateral sclerosis. Amyotroph Lateral Scler Front Degener 21:18–27. 10.1080/21678421.2020.1837176 10.1080/21678421.2020.1837176 33602015

[20] Caminiti SP, Sala A, Presotto L et al (2021) Validation of FDG-PET datasets of normal controls for the extraction of SPM-based brain metabolism maps. Eur J Nucl Med Mol Imaging 48:2486–2499. 10.1007/s00259-020-05175-1 33423088 10.1007/s00259-020-05175-1

[21] Della Rosa PA, Cerami C, Gallivanone F et al (2014) A standardized [18F]-FDG-PET template for spatial normalization in statistical parametric mapping of dementia. Neuroinformatics 12:575–593. 10.1007/s12021-014-9235-4 24952892 10.1007/s12021-014-9235-4

[22] Mayka MA, Corcos DM, Leurgans SE, Vaillancourt DE (2006) Three-dimensional locations and boundaries of motor and premotor cortices as defined by functional brain imaging: a meta-analysis. Neuroimage 31:1453–1474. 10.1016/j.neuroimage.2006.02.004 16571375 10.1016/j.neuroimage.2006.02.004 PMC2034289

[23] Fan L, Li H, Zhuo J et al (2016) The Human Brainnetome Atlas: a new brain atlas based on connectional architecture. Cereb Cortex 26:3508–3526. 10.1093/cercor/bhw157 27230218 10.1093/cercor/bhw157 PMC4961028

[24] Gastaut JL, Bartolomei F (1994) Mills’ syndrome: ascending (or descending) progressive hemiplegia: a hemiplegic form of primary lateral sclerosis? J Neurol Neurosurg Psychiatry 57:1280–1281. 10.1136/jnnp.57.10.1280 7931406 10.1136/jnnp.57.10.1280 PMC485513

[25] Fernandes PM, Turner MR, Zeidler M et al (2015) Progressive hemiparesis in a 75-year-old man. Pract Neurol 15:63–71. 10.1136/practneurol-2014-000950 25253896 10.1136/practneurol-2014-000950

[26] Younger DS, Chou S, Hays AP et al (1988) Primary lateral sclerosis: a clinical diagnosis reemerges. Arch Neurol 45:1304–1307. 10.1001/archneur.1988.00520360022005 3196189 10.1001/archneur.1988.00520360022005

[27] Finegan E, Chipika RH, Li Hi Shing S et al (2019) The clinical and radiological profile of primary lateral sclerosis: a population-based study. J Neurol 266:2718–2733. 10.1007/s00415-019-09473-z 31325016 10.1007/s00415-019-09473-z

[28] Tartaglia MC, Laluz V, Rowe A et al (2009) Brain atrophy in primary lateral sclerosis. Neurology 72:1236–1241. 10.1212/01.wnl.0000345665.75512.f9 19349603 10.1212/01.wnl.0000345665.75512.f9

[29] Butman JA, Floeter MK (2007) Decreased thickness of primary motor cortex in primary lateral sclerosis. Am J Neuroradiol 28:87–91 17213431 PMC8134097

[30] Smith CD (2002) Serial MRI findings in a case of primary lateral sclerosis. Neurology 58:647–649. 10.1212/wnl.58.4.647 11865149 10.1212/wnl.58.4.647

[31] King A, Curran O, Al-Sarraj S (2013) Atypical progressive supranuclear palsy presenting as primary lateral sclerosis. J Neurol Sci 329:69. 10.1016/j.jns.2013.03.015 23570981 10.1016/j.jns.2013.03.015

[32] de Boer EMJ, de Vries BS, Van Hecke W et al (2024) Diagnosing primary lateral sclerosis: a clinico-pathological study. J Neurol 272:46. 10.1007/s00415-024-12816-0 39666071 10.1007/s00415-024-12816-0

[33] Eisen A, Kim S, Pant B (1992) Amyotrophic lateral sclerosis (ALS): a phylogenetic disease of the corticomotoneuron? Muscle Nerve 15:219–224. 10.1002/mus.880150215 1549143 10.1002/mus.880150215

[34] Hudson AJ, Kiernan JN (1988) Preservation of certain voluntary muscles in motoneurone disease. Lancet 331:652–653. 10.1016/S0140-6736(88)91455-9 10.1016/s0140-6736(88)91455-9 2894586

[35] Dadon-Nachum M, Melamed E, Offen D (2011) The “Dying-Back” phenomenon of motor neurons in ALS. J Mol Neurosci 43:470–477. 10.1007/s12031-010-9467-1 21057983 10.1007/s12031-010-9467-1

[36] Zhai P, Pagan F, Statland J et al (2003) Primary lateral sclerosis: a heterogeneous disorder composed of different subtypes? Neurology 60:1258–1265. 10.1212/01.wnl.0000058900.02672.d2 12707427 10.1212/01.wnl.0000058900.02672.d2

[37] Zimmer ER, Parent MJ, Souza DG et al (2017) [18F]FDG PET signal is driven by astroglial glutamate transport. Nat Neurosci 20:393–395. 10.1038/nn.4492 28135241 10.1038/nn.4492 PMC5378483

[38] Xiang X, Wind K, Wiedemann T et al (2021) Microglial activation states drive glucose uptake and FDG-PET alterations in neurodegenerative diseases. Sci Transl Med 13:eabe5640. 10.1126/scitranslmed.abe5640 34644146 10.1126/scitranslmed.abe5640

[39] Turner MR, Hammers A, Al-Chalabi A et al (2007) Cortical involvement in four cases of primary lateral sclerosis using [11C]-flumazenil PET. J Neurol 254:1033–1036. 10.1007/s00415-006-0482-7 17294065 10.1007/s00415-006-0482-7

[40] Paganoni S, Alshikho MJ, Zürcher NR et al (2018) Imaging of glia activation in people with primary lateral sclerosis. NeuroImage Clin 17:347–353. 10.1016/j.nicl.2017.10.024 29159046 10.1016/j.nicl.2017.10.024 PMC5681341

[41] Alshikho MJ, Zürcher NR, Loggia ML et al (2018) Integrated magnetic resonance imaging and [11 C]-PBR28 positron emission tomographic imaging in amyotrophic lateral sclerosis. Ann Neurol 83:1186–1197. 10.1002/ana.25251 29740862 10.1002/ana.25251 PMC6105567

[42] Berti V, Mosconi L, Pupi A (2014) Brain: normal variations and benign findings in fluorodeoxyglucose-PET/computed tomography imaging. PET Clin 9:129–140. 10.1016/j.cpet.2013.10.006 24772054 10.1016/j.cpet.2013.10.006 PMC3998066

[43] Knopman DS, Jack CR, Wiste HJ et al (2014) 18F-fluorodeoxyglucose positron emission tomography, aging, and apolipoprotein E genotype in cognitively normal persons. Neurobiol Aging 35:2096–2106. 10.1016/j.neurobiolaging.2014.03.006 24702820 10.1016/j.neurobiolaging.2014.03.006 PMC4053507

