# Supplementary material for: Inconsistent primary motor cortex glucose hypometabolism in primary lateral sclerosis
Source: J Neurol. 2025 May 20;272(6):410. doi: 10.1007/s00415-025-13089-x (PMC12092561; doi:10.1007/s00415-025-13089-x)
Supplement: Supplementary file 1 — Supplementary file1 (PDF 415 kb) [file 415_2025_13089_MOESM1_ESM.pdf]

Inconsistent primary motor cortex glucose hypometabolism in primary lateral sclerosis

Journal of Neurology

Annaliis Lehto[a,b], Julia Schumacher[b,c], Jens Kurth[d], Bernd J Krause[d], Elisabeth Kasper[b,c], Stefan Teipel[b,e], Johannes Prudlo[b,c*] and for the Alzheimer's Disease Neuroimaging Initiative[1]

[a] Translational Neurodegeneration Section "Albrecht Kossel", Department of Neurology, Rostock University Medical Center, Rostock, Germany,
[b] Deutsches Zentrum für Neurodegenerative Erkrankungen (DZNE), Rostock-Greifswald, Rostock, Germany,
[c] Department of Neurology, Rostock University Medical Center, Rostock, Germany,
[d] Department of Nuclear Medicine, Rostock University Medical Center, Rostock, Germany,
[e] Department of Psychosomatic Medicine, Rostock University Medical Center, Rostock, Germany

Corresponding Author:
Johannes Prudlo, M.D.
E-mail: johannes.prudlo@med.uni-rostock.de

[1] Data used in preparation of this article were obtained from the Alzheimer's Disease Neuroimaging Initiative (ADNI) database (adni.loni.usc.edu). As such, the investigators within the ADNI contributed to the design and implementation of ADNI and/or provided data but did not participate in analysis or writing of this report. A complete listing of ADNI investigators can be found at: http://adni.loni.usc.edu/wp-content/uploads/how_to_apply/ADNI_Acknowledgement_List.pdf

	Time	Duration	Affected regions	M1_R	M1_L	hf_L	hf_R	ul_L	ul_R	tl_L	tl_R	ll_R	ll_L	SMA_R	SMA_L	preSMA_R	preSMA_L	PMd_R	PMd_L	PMv_R	PMv_L
pat1	1	54	BUL, UL, LL	0.90	1.70	0.20	-1.19	0.76	1.31	-1.92	-1.55	1.78	1.66	2.29	0.91	0.78	0.67	1.83	2.73	-1.74	-1.29
	2	101	BUL, UL, LL	-3.16	-3.32	-3.04	-3.34	-2.24	-1.44	-0.06	-1.67	-1.78	-1.27	-1.93	-2.25	-0.35	-0.43	-1.05	-0.24	-3.40	-2.95
	3	125	BUL, UL, LL	-3.22	-3.52	-3.77	-3.58	-2.55	-2.60	-0.69	-2.00	-0.24	-1.28	-1.39	-2.95	-1.54	-2.13	-1.38	-1.94	-4.21	-4.20
pat2	1	14	BUL	-0.78	-0.45	-0.22	0.72	-0.27	-0.51	-0.22	2.42	-0.35	0.34	0.29	0.89	0.49	0.31	0.82	0.01	2.99	-0.05
	2	26	BUL	-0.35	-0.33	0.33	0.80	-0.21	-0.39	0.63	2.49	-0.16	1.02	0.74	1.37	-0.24	-0.15	0.65	0.08	2.40	-0.45
	3	48	BUL, UL	-0.94	-0.69	0.41	0.99	-0.67	-0.73	0.83	2.59	-0.24	0.78	0.52	1.28	0.38	0.59	0.55	0.19	2.60	-0.01
pat3	1	7	LL, UL	-2.32	-1.11	0.47	0.00	-0.35	-1.53	0.82	0.18	-0.33	-0.20	-1.35	-0.43	-0.46	-0.02	-1.19	0.74	0.00	1.07
	2	24	LL, UL, BUL	-3.17	-2.65	-0.36	0.02	-1.71	-3.33	2.05	2.30	-1.29	-0.57	-3.14	-1.54	-1.00	-0.50	-2.40	0.18	0.36	1.56
pat4	1	21	BUL, UL, LL	-1.64	-2.10	-1.44	-0.97	-1.28	-1.63	0.40	0.14	-1.34	-1.05	-1.58	-1.02	-1.64	-1.22	-1.77	-0.61	-0.95	-0.18
	2	32	BUL, UL, LL	-1.28	-2.60	-1.63	-0.78	-1.76	-1.03	0.60	0.76	-1.33	-0.93	-1.25	-0.93	-1.18	-0.75	-1.30	-1.12	-0.75	-1.23
pat5	1	26	LL, UL, BUL	-0.15	1.92	1.64	0.82	0.47	0.11	0.49	3.52	-0.51	0.22	0.16	1.13	0.69	0.56	0.08	1.21	3.48	3.72
	2	41	LL, UL, BUL	0.32	2.02	1.41	1.67	0.35	0.59	0.72	2.76	-0.15	0.37	0.85	1.50	1.29	1.30	0.34	1.74	4.20	3.48
pat6	1	39	LL	0.35	-1.09	-0.50	2.04	-0.78	0.13	-1.39	-2.26	-1.85	-2.63	-2.12	-3.39	-0.33	-1.01	-0.73	-2.55	0.78	-1.14
pat7	1	56	BUL, UL, LL	0.03	0.17	1.30	1.58	-0.67	0.65	0.69	0.30	-0.89	-1.02	-0.42	-0.07	-0.17	0.37	1.46	0.81	2.04	2.38
pat8	1	65	LL, UL, BUL	0.13	-0.49	0.98	0.27	0.21	0.67	-0.38	-0.07	-1.76	-0.79	-0.95	-0.02	-0.35	0.54	1.64	2.65	2.19	2.06
pat9	1	74	LL, UL, BUL	-1.72	-0.03	0.90	1.17	-0.14	-1.33	1.74	1.08	-0.27	0.36	-0.18	0.21	0.43	0.54	-0.61	-0.29	2.28	3.13

Supplementary table 1. Regional w-scores of PLS patients

Duration is given in months and affected regions entail regions where patients report noticeable symptomatic involvement. W-scores between -1.65 and -1.96 are shown in light orange (one-sided p-values between 0.05 and 0.025), and w-scores < -1.96 are shown in dark orange (one-sided p-value < 0.025). Right and left refer to the assessed hemisphere. BUL, bulbar; hf, head and face; L, left; ll, lower limb; M1, primary motor cortex; PMd, dorsal pre-motor cortex; PMv, ventral pre-motor cortex; R, right; SMA, supplementary motor area; tl, throat and larynx; ul, upper limb.
